# The Editor's Letter-Box

**Published:** 1915-07-31

**Authors:** P. J. Hartog

**Affiliations:** Academic Registrar. South Kensington, London, S.W.


(3) University of London.

Dear Sir,—I regret to inform you that, after consideration of your letter of June 19 last, making application for the temporary recognition of the West London Hospital for clinical study for the purposes of the M.B., B.S. examination, and of reports thereon from the relevant committees of the Senate, it was resolved by the Senate at their meeting on July 14, 1915, that the application be not acceded to.—I am, dear Sir, yours faithfully,

(Signed) P. J. Hartog, Academic Registrar.

South Kensington, London, S.W.
July 15, 1915.

